# Peptide vaccines: an innovative therapeutic approach against antibiotic-resistant bacterial infections

**DOI:** 10.3389/fimmu.2025.1567584

**Published:** 2025-05-21

**Authors:** Fatemeh Tavassoli Razavi, Nasrin Salari, Atena Emami, Dariush Haghmorad, Rasoul Baharlou

**Affiliations:** Department of Immunology, School of Medicine, Semnan University of Medical Sciences, Semnan, Iran

**Keywords:** peptide vaccines, *Enterococcus faecium*, methicillin-resistant *Staphylococcus aureus*, *Klebsiella pneumoniae*, *Acinetobacter baumannii*, *Pseudomonas aeruginosa*, *Enterobacter* spp

## Abstract

Bacterial infections continue to pose a serious threat to global health, especially with the growing challenge of multidrug-resistant pathogens. While traditional vaccines have been pivotal in reducing disease burden, they come with limitations such as variable efficacy, safety concerns, and limited ability to address the diversity of bacterial strains. This review highlights the promise of peptide-based vaccines as an innovative approach to overcoming these hurdles. By targeting specific regions of bacterial proteins, peptide vaccines can elicit precise immune responses with improved safety and broader applicability. Advances in technology, including bioinformatics and delivery systems, have enhanced their design, making them more stable, effective, and easier to produce. These vaccines work by activating both antibody and T-cell responses through well-defined mechanisms. Different types, such as linear peptides, cyclic peptides, and synthetic long peptides, offer diverse strategies to tailor immune protection. The role of adjuvants and advanced delivery methods, like nanoparticles and liposomes, further improve their potential. Exciting progress has been made against the ESKAPE pathogens — *Enterococcus faecium*, *Staphylococcus aureus*, *Klebsiella pneumoniae*, *Acinetobacter baumannii*, *Pseudomonas aeruginosa*, and *Enterobacter* spp. Peptide vaccines offer a forward-thinking, adaptable solution to reduce bacterial infections and mitigate the rise of antibiotic resistance, paving the way for safer and more effective prevention strategies. This review underscores the critical role of peptide-based vaccines in combating bacterial infections, advocating for ongoing research to unlock their full potential.

## 1 Introduction

Bacterial infections remain a major global health problem, causing substantial morbidity and mortality; notably in low- and middle-income countries (LMICs) with challenging healthcare system resources (1, 2). Tens of millions die from bacterial diseases, including respiratory infections caused by tuberculosis and pneumonia (3). These infections predominantly affect young children, elder people and people with compromised immune systems (4). Among the top five global causes of death are lower respiratory tract infections (LRTIs); pneumonia is the most common (5). Along with pneumonia, other bacterial infections—such as bloodstream infections (sepsis), urinary tract infections (especially catheter-associated), and bacterial meningitis—contribute significantly to global mortality, particularly when caused by multidrug-resistant organisms (6).

Vaccination remains one of the most effective public health interventions to prevent bacterial infections. Vaccines prime the immune system, enabling it to recognize and respond to pathogens before an infection can be established (7). Conventional vaccines — live-attenuated, inactivated, and subunit vaccines — have significantly decreased the incidence of various bacterial diseases. However, these approaches have intrinsic limitations. For instance, although live-attenuated vaccines are highly immunogenic, they pose safety concerns since the attenuated strains can revert to a virulent form, particularly in the immunocompromised host (8–10). Inactivated vaccines are safer but they often trigger only moderate immune responses, requiring the use of adjuvants or booster doses (9, 10). Subunit vaccines, which employ purified pathogen components, frequently fail to generate durable immunity (10, 11). Additionally, the diversity of bacterial antigens is a major problem. Traditional vaccines are often strain- or serotype-specific, limiting their broad use (12).

The increase in multidrug-resistant (MDR) bacteria highlights the need for new vaccines. Antibiotic resistance has reached a global crisis, with MDR pathogens such as methicillin-resistant *Staphylococcus aureus* (MRSA) and *carbapenem-resistant Enterobacteriaceae*, emerging as major public health threats (13). These so-called “superbugs” (14) are particularly alarming as they cause hard-to-treat infections, leading to increased morbidity, mortality, and healthcare costs (15).

In addition to their resistance to antibiotics, MDR bacteria have evolved multiple immune evasion strategies that enable persistence and pathogenicity (16). These include biofilm formation, which creates a physical barrier that impedes phagocytosis and limits immune cell access (17); secretion of immunomodulatory toxins, such as leukocidins and hemolysins, which damage immune cells or disrupt cytokine signaling (18); antigenic variation, involving changes in surface antigens that help bacteria escape recognition by antibodies and T-cells (19); inhibition of phagocytosis, via surface proteins (e.g., protein A in *S. aureus*) that bind antibodies in a non-opsonizing orientation (20); and downregulation of antigen presentation, where bacterial factors interfere with major histocompatibility complex (MHC)" expression, limiting T-cell activation (21). These sophisticated strategies enable pathogens to evade both innate and adaptive immunity, establish chronic infections, and challenge vaccine-mediated protection. Consequently, there is an imperative to develop innovative vaccine platforms that not only prevent infection but also reduce antibiotic reliance and effectively counter bacterial immune evasion strategies.

The bacterial peptide-based vaccine is a new paradigm of bacterial immunization that uses short sequences of amino acids from pathogen-specific antigens to elicit a targeted response against specific epitopes (the portions of antigens that the immune system uses to recognize pathogens). Peptide vaccines bypass risks associated with using whole pathogens, such as reversion to virulence, associated with traditional approaches. Peptide vaccines targeting conserved protein regions critical to bacterial survival and less prone to mutation offer the possibility of broad immunity and can effectively combat antigenic variation (22). New insights from bioinformatics and proteomics have played a major role in discovering immunogenic peptides. This allows the rational design of vaccines against individual pathogens or multiple strains (23). In addition to providing immunological advantages, peptide vaccines offer significant logistical and economic benefits. Their synthetic derivation makes them easy to manufacture, which lowers production costs and simplifies storage and distribution (10). Innovative delivery systems, such as nanoparticle-based carriers, enhance peptide stability and facilitate targeted endocytic uptake by antigen-presenting cells. This promotes efficient processing and presentation of epitopes on MHC class I and II molecules, thereby eliciting robust humoral and cellular immune responses and optimizing overall vaccine immunogenicity and efficacy (24, 25).

In general, peptide-based vaccines present a promising, flexible approach to overcoming many of the challenges faced by traditional bacterial vaccines. Offering a targeted, customizable, and potentially safer solution, they have the potential to significantly reduce the global impact of bacterial infections, especially as antibiotic resistance continues to rise. Continued research into peptide immunization is essential to harness their full potential in creating effective, lasting vaccines against a wide range of bacterial pathogens. In this review, we examined the benefits and future approaches of peptide vaccines.

## 2 Mechanisms of peptide vaccines in immunity against bacterial infections

### 2.1 Immunological basis of peptide vaccines

**Figure 1:**
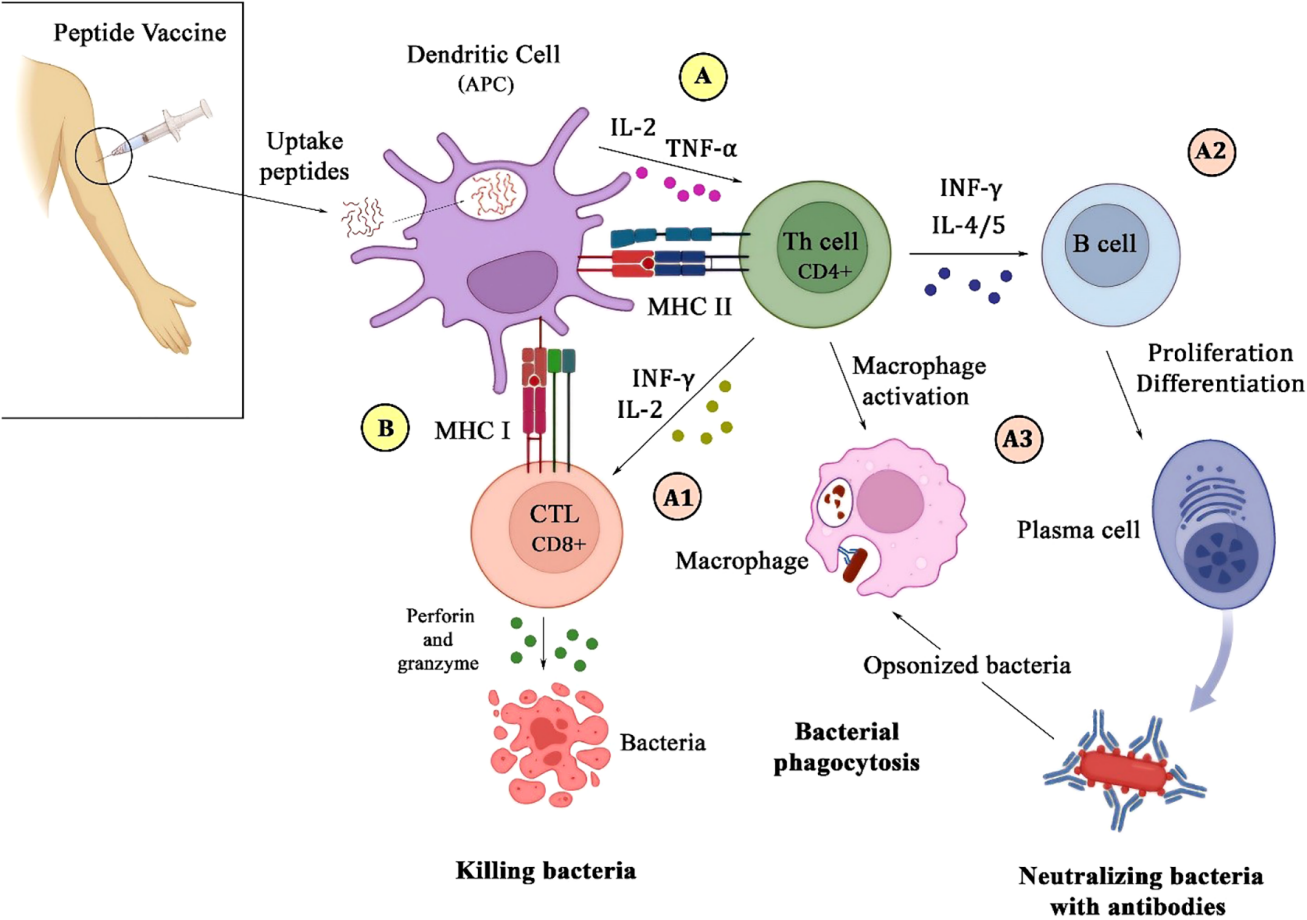
Schematic illustration of the immune response to a peptide vaccine. Upon injection of the vaccine, microbial peptides are internalized by dendritic cells, which then process and present them via MHC class II **(A)** and I **(B)** molecules to CD_4_^+^ and CD_8_^+^ T-cells. This interaction leads to the secretion of IL-2 and TNF-α by DC on CD_4_^+^ T-cells **(A)**. Activated CD_4_^+^ T-cells enhance CD_8_^+^ T-cells function by secreting IFN-γ and IL-2, which promote bacterial killing through perforin and granzyme release **(A1)**. Additionally, CD_4_^+^ T-cells stimulate B-cells to differentiate into plasma cells to produce antibodies **(A2)**, and also activate macrophages to recognize and phagocytose antibody-opsonized bacteria **(A3)**.

Peptide vaccines offer several advantages, including precise antigen targeting, favorable safety profiles, and the ability to induce both humoral and cellular immune responses (22). The fundamental mechanism underlying their immunogenicity involves the presentation of antigenic peptides by MHC molecules to T-cells, thereby initiating an adaptive immune response against bacterial pathogens (**Figure 1**) (26).

#### 2.1.1 Antigen presentation by MHC molecules and T-cell activation

These studies generally design peptide vaccines that are composed of selected amino acid sequences, termed epitopes, which correspond to the immunogenic regions of bacterial antigens. Following administration, such epitopes are internalized by antigen-presenting cells, mainly dendritic cells (27). The cell processes the peptides and then presents them on the surface in association with MHC molecules. MHC class I and II present peptides to cytotoxic CD_8_^+^ T and CD_4_^+^T helper cells, which play a crucial role in coordinating the immune response through the elimination of intracellular pathogens and secretion of cytokines that support both cellular and humoral immunity (28). Dendritic cells, along with the presentation of peptides with MHC class II, secrete interleukin 2 and TNF-α on CD_4_^+^T to activate it (29). The efficacy of peptide vaccines is strongly influenced by the ability of the epitopes to bind to MHC molecules, which promotes T-cell activation and a potent immune defense against bacterial infections.

#### 2.1.2 Stimulation of humoral and cellular immune responses

A well-designed vaccine should trigger both humoral and cellular immune responses to provide comprehensive protection against bacterial pathogens. Activated CD_4_^+^T activates B-cells by secreting INF-γ and interleukins such as 4 and 5. The humoral immune response, driven by B-cell activation and antibody production, is essential for neutralizing extracellular bacteria and preventing colonization. The antibodies can neutralize bacterial toxins, inhibit bacterial adhesion to host tissues, and facilitate opsonization, marking bacteria for phagocytosis (29, 30). Cellular immunity, primarily mediated by T-cells, plays a crucial role in the defense against intracellular pathogens. Cytotoxic CD_8_^+^ T-cells recognize and eliminate infected cells, and CD_4_^+^ helper T-cells stimulate phagocytic activity and also enhance the responses of cytotoxic T-cells by secreting INF-γ and interleukin 2, contributing to the development of lasting immunity (28, 29). Peptide vaccines aim to induce a comprehensive and durable immune response against bacterial infections by targeting epitopes that engage both humoral and cellular immunity.

### 2.2 Types of peptide vaccines

Peptide vaccines are classified based on the structure of the peptide as well as its targeted immune response. There are a few categories of peptides, such as linear peptides, cyclic peptides, epitope-based peptides, and synthetic long peptides (SLPs). Different types have distinct advantages for bacterial vaccine development.

#### 2.2.1 Linear vs. cyclic peptides

Most peptide vaccines are based on linear epitopes—short, sequential amino acid fragments derived from pathogenic proteins. These are relatively easy to identify, synthesize, and screen using high-throughput platforms. However, linear peptides often fail to replicate the native three-dimensional structure of proteins, leading to reduced stability and suboptimal immune recognition *in vivo*. In contrast, conformational epitopes—formed by amino acid residues brought together by protein folding—are typically more immunogenic as they better resemble the native surface of pathogens recognized by B-cell receptors. Peptide vaccines face a fundamental limitation in effectively presenting these conformational structures due to the inherent flexibility and lack of structural complexity of linear peptides. Cyclic peptides, by forming stable and rigid ring structures, can mimic conformational epitopes more accurately. Their restricted conformational mobility enhances binding specificity to MHC molecules and immune receptors, and increases resistance to enzymatic degradation. Thus, employing cyclic peptides in vaccine design can partially overcome the challenge of inducing immune responses against conformational epitopes, though reproducing full protein structures remains difficult in synthetic constructs (31–33).

#### 2.2.2 Epitope-based peptides

Epitope-based vaccines are composed of peptides representing either T-cell or B-cell epitopes. T-cell epitopes are selected based on their ability to bind MHC molecules, leading to the activation of cytotoxic T lymphocyte (CTL) or helper T-cell responses that are essential for combating intracellular pathogens. In contrast, B-cell epitopes—often derived from surface-exposed regions of bacterial proteins—aim to induce robust antibody responses, which are critical for neutralizing extracellular pathogens (34, 35). To enhance immunogenic breadth, multiple epitopes can be combined into a single construct, resulting in multiepitope-based peptide vaccines (36). These constructs are designed through a rational, immunoinformatics-driven process involving the prediction and selection of highly antigenic, non-toxic, and non-allergenic epitopes with broad population coverage. Then, the selected epitopes are assembled using appropriate peptide linkers and, in some cases, fused to molecular adjuvants or carrier sequences to enhance immunogenicity (37). The choice of linker is critical for maintaining proper antigen processing and structural integrity. Flexible linkers such as AAY or KK are often used to join CTL and B-cell epitopes due to their capacity to promote proteasomal cleavage and enhance epitope presentation. However, their high conformational mobility may result in reduced structural integrity, unwanted interactions between adjacent epitopes, or impaired biological activity. On the other hand, rigid linkers like EAAAK provide fixed spatial separation between functional domains, helping to maintain proper folding and minimize domain interference but may hinder the conformational flexibility needed for antigen processing by APCs. Thus to ensure optimal structural conformation and antigen processing, peptide linkers are chosen based on the structural and immunological demands of the vaccine construct (37–39). Following design, the synthetic gene encoding the multi-epitope construct is codon-optimized and recombinantly expressed in suitable host systems. The suitability of these vaccine candidates depends on several critical parameters, including physicochemical stability, expression yield, proper folding, epitope accessibility, and cost-effectiveness of production. (37). Multiepitope vaccines have demonstrated promise against antigenically diverse or drug-resistant pathogens—such as ESKAPE bacteria—by eliciting multi-faceted immune responses and potential cross-protection.

#### 2.2.3 Synthetic long peptides

SLPs are longer peptides, 20 to 35 amino acids long, that contain multiple T-and B-cell epitopes within a single structure. This enables the activation of both arms of the immune system. SLPs often have multiple MHC-binding sites, enabling a broader T-cell response and increased potential for cross-reactivity. Their design imitates natural antigens in a way that enhances antigen presentation and immune recognition. These features make SLPs promising for inducing strong and lasting immunity in bacterial vaccine development (40, 41).

### 2.3 Adjuvants and delivery systems

To enhance the immunogenicity of peptide vaccines, adjuvants and advanced delivery systems are often used. Adjuvants amplify the immune response to an antigen, while delivery systems control the release of the vaccine and target its delivery, overcoming the low immunogenicity of isolated peptide antigens (42).

#### 2.3.1 Adjuvants in enhancing immunogenicity

Adjuvants stimulate immune cells and prolong antigen exposure, thereby enhancing vaccine efficacy. Traditional adjuvants, such as alum, play a major role in promoting antibody responses. However, newer adjuvants, including Toll-like receptor (TLR) agonists, saponins, and liposomes, are designed to boost cellular immunity by activating dendritic cells and promoting antigen presentation. In peptide vaccines, adjuvants are particularly valuable as they provide “danger signals” that amplify the immune response (42, 43). Despite their efficacy, the use of adjuvants comes with potential risks, particularly concerning their safety. Overuse or inappropriate selection of adjuvants can result in allergic reactions and, in some cases, autoimmune disorders. For example, TLR agonists may overstimulate the immune system, leading to unwanted inflammatory responses or hypersensitivity, especially in vulnerable individuals. Additionally, some adjuvants, such as saponins, have been linked to local reactions and systemic toxicity. These risks necessitate careful optimization and controlled usage of adjuvants in peptide vaccines to prevent any adverse effects (44, 45).

#### 2.3.2 Delivery systems: nanocarriers, liposomes, and conjugate vaccines

New delivery systems, including nanocarriers and liposomes, have been developed that protect peptides from degradation, control their release rate, and target the immune cells with high precision. Nanocarriers, consisting of nanoparticles and polymer systems, can encapsulate peptides for protection and assured delivery to the APCs. Nanoparticles may be prepared as pathogen imitations that can be taken up by dendritic cells at enhanced rates, therefore promoting antigen presentation (46). Since liposomes are vesicular carriers of lipids, they would, in turn, encapsulate the peptides for longer circulation and specific delivery to immune cells. They also act as an adjuvant and enhance humoral and cellular responses (47). Conjugate vaccines link peptides to carrier proteins such as tetanus or diphtheria toxoids. They enhance immunogenicity by providing extra T-cell epitopes. This approach is quite useful in groups where immune responses are generally poor, such as older adults and young children (48).

Despite their advantages, each delivery modality carries specific safety concerns. Nanocarriers may induce dose−dependent cytotoxicity, oxidative stress, and pro−inflammatory cytokine release—effects that vary with particle size, surface charge, and composition, and can lead to tissue damage or systemic inflammation (49). Liposomes are prone to complement activation−related pseudo-allergy (CARPA), manifesting as infusion−related hypersensitivity (flushing, dyspnea, hypotension) in susceptible individuals (50). Conjugate vaccines, while generally well tolerated, can cause local injection−site reactions and, in rare cases, carrier−induced epitope suppression, whereby pre−existing immunity to the protein carrier (e.g., tetanus toxoid) diminishes the response to the linked peptide antigen (51, 52).

## 3 Peptide vaccines against bacterial infections

As mentioned, peptide vaccines have emerged as a therapeutic strategy by targeting specific antigenic determinants, which can induce precise and potent immune responses against a wide range of bacterial pathogens in the fight against bacterial infections. One of the most promising strategies in this context involves the use of conserved regions of bacterial proteins. These conserved sequences are shared across multiple strains or species of a pathogen and are less prone to mutation, making them ideal targets for vaccine development. Targeting such regions ensures broader coverage against diverse bacterial variants, including multidrug-resistant strains. Furthermore, these proteins are predicted to be highly antigenic and essential for pathogen survival and reduces the risk of immune escape due to antigenic variation. Moreover, peptides derived from conserved epitopes are more likely to elicit cross-protective immune responses, which is crucial for combating the genetic variability characteristic of ESKAPE pathogens (53, 54).

This review analyzes the development of peptide vaccines for the ESKAPE pathogens — *Enterococcus faecium*, *Staphylococcus aureus*, *Klebsiella pneumoniae*, *Acinetobacter baumannii*, *Pseudomonas aeruginosa* and *Enterobacter* spp. — each of which presents unique challenges in immunogenicity and pathogen persistence. Peptide vaccines in development against ESKAPE are shown in **Table 1**.

**Table 1:** Peptide vaccines in development against ESKAPE.

Bacterium	Target(s) of Peptide Vaccine	Development Stage	Source(s)
*Streptococcus pneumoniae*	PspA, PhtD PspA, PhtD, PspC PspA, CbpA, PhtD, PiuA	Preclinical Preclinical Preclinical	(55) (56) (57)
*Streptococcus pneumoniae*	PspC	In silico	(58)
*Streptococcus pneumoniae*	PhtD, PcpA PcpA, PhtD, PlyD1 dPly, PhtD	Clinical trials - Phase I Clinical trials - Phase I Clinical trials - Phase I & II	(59) (60) (61, 62)
*Staphylococcus aureus*	ClfA, ClfB R13	Preclinical Preclinical	(63) (64)
*Staphylococcus aureus*	MABC, NABC, and PIc	Preclinical	(65)
*Staphylococcus aureus*	Alpha-enolase, ClfA, IsdB FnBPA, FnBPB Glycosyltransferase, EBP, Staphylococcal secretory antigen	In silico In silico In silico	(66) (67) (68)
*Staphylococcus aureus*	ClfB, FnbpA, Hla, IsdA, IsdB, LukE, SdrD, and SdrE	In silico & Preclinical	(53, 69)
*Staphylococcus aureus*	rFSAV IsdB	Clinical trial - Phase II Clinical trial - Phase II	(70) (71)
*Pseudomonas aeruginosa*	OprF, OprI	Clinical trials - Phase II & III Clinical trials - Phase II Clinical trials - Phase I	(72) (73) (74)
*Pseudomonas aeruginosa*	PilA PBD	Preclinical Preclinical	(75) (76)
*Pseudomonas aeruginosa*	OprF, OprE fructose bisphosphate aldolase (FBA)	In silico In silico	(77) (78)
*Enterococcus faecium* (VRE)	PBP5 Psts	In silico In silico	(79) (80)
*Klebsiella pneumoniae* (CRKP)	OmpA, OmpW, and FepA (mHla-EpiVac) P40	Preclinical Preclinical	(81) (82)
*Klebsiella pneumoniae* (CRKP)	type 3 fimbrial protein OMPK17 FepA	In silico In silico In silico	(83) (84) (85)
*Acinetobacter baumannii*	Ata, FilF, and Nucab Ata	Preclinical Preclinical	(86) (87)
*Acinetobacter baumannii*	EpsA, CsuB FilF	In silico In silico	(88) (89)

### 3.1 *Streptococcus pneumoniae* (pneumococcus)

*Streptococcus pneumoniae* remains a significant global health burden, causing a range of severe infections, including pneumonia, meningitis, and sepsis. While pneumococcal conjugate vaccines (PCVs) have significantly reduced the incidence of pneumococcal disease, serotype diversity and the emergence of non-vaccine serotypes limit their effectiveness (90, 91).

Peptide vaccines offer a promising alternative by targeting conserved antigens present across multiple pneumococcal strains. This approach has the potential to provide broader protection against a wider range of serotypes, including those not covered by current vaccines. PspA (Pneumococcal Surface Protein A) is the most promising vaccine candidate (92). This protein—along with several other surface proteins—is highly conserved across pneumococcal strains and is recognized by both B and T-cells. Recently Bahadori et al. demonstrated the efficacy of the fusion PhtD-PspA-PspC-based peptide vaccine in inducing protective antibody responses and improving survival in animal models of pneumococcal infection in a preclinical study (56). Researchers have also conducted clinical trials combining the peptide vaccine and the pneumococcal conjugate vaccine for greater efficacy (60–62). However the multiepitope peptide vaccine against *Streptococcus pneumoniae* showed limited efficacy in a clinical trial for otitis media, likely due to antigenic polymorphism among strains. The selected epitopes did not cover the full antigenic diversity, resulting in weak cross-protection. Additionally, synthetic peptides may fail to replicate the native structure of full-length proteins, reducing their immunogenicity (62). Preclinical and clinical trials are still ongoing in this field, and if successful, peptide vaccines could provide a valuable tool in the fight against pneumococcal disease, providing broader protection and overcoming the limitations of current vaccine strategies.

### 3.2 Staphylococcus aureus

*Staphylococcus aureus*, particularly *methicillin-resistant S. aureus* (MRSA), remains a significant cause of hospital-acquired infections worldwide. Its ability to form biofilms, evade host immune responses, and develop resistance to multiple antibiotics has made it a challenging pathogen to combat (93).

Peptide-based vaccines represent a promising approach to combat *Staphylococcus aureus* infections by targeting key virulence factors to elicit robust immune responses and prevent bacterial colonization. Notably, clumping factors A and B (ClfA and ClfB) have emerged as important targets, as explored by Dey et al. ClfA facilitates bacterial adhesion to host tissues, while ClfB promotes attachment to nasal corneocytes and triggers human platelet aggregation. These antigens are both highly immunogenic and conserved, making them suitable candidates for vaccine development, especially against multidrug-resistant strains (63). Also, the newest study developed a peptide vaccine using B and T-cell epitopes from MABC, NABC, and PIc proteins to combat *Staphylococcus aureus*. Mice immunized with this vaccine showed the best skin lesion healing, with high IgG levels, increased INF-γ, and enhanced CD_4_/CD_8_ T-cell counts. This approach improved both humoral and cellular immunity, demonstrating promising results for *S. aureus* vaccine development (65).

Several multi-antigenic peptide vaccines have been evaluated in preoperative settings. For example, the V710 vaccine targeting IsdB did not reduce the incidence of postoperative infections in cardiac surgery patients and was paradoxically associated with increased mortality in those who became infected—likely due to insufficient preexisting IL-2 and IL-17 responses (71, 94). Conversely, the rFSAV vaccine, which incorporates five different antigens, demonstrated good safety and strong, rapid humoral responses in a phase II clinical trial among patients undergoing elective orthopedic surgery (70). These findings highlight the challenges inherent in developing effective vaccines against *S. aureus*, including the need for appropriate antigen selection and consideration of host immune profiles.

Despite these findings, no peptide-based *S. aureus* vaccine has yet reached clinical application, underscoring the need for further experimental and clinical investigations (95).

### 3.3 Pseudomonas aeruginosa

*Pseudomonas aeruginosa* is a versatile opportunistic pathogen that can cause a wide range of infections, particularly in immunocompromised individuals. Its intrinsic antibiotic resistance and ability to form biofilms make it a significant challenge in healthcare settings (96).

Peptide vaccines represent a promising strategy to combat *P. aeruginosa* infections. Key targets for peptide-based vaccines against *P. aeruginosa* include outer membrane proteins (OMPs) and also the receptor-binding domains (RBD) of pili. Studies to develop a peptide vaccine against RBD have been conducted in the past (97, 98). Recently, in the study by Adlbrecht et al., a peptide vaccine against OprI and OprF was evaluated in phase II and III clinical trials, and it showed that while the vaccine was highly immunogenic, it did not reduce mortality from *Pseudomonas aeruginosa* (72). Additionally, Roy et al. designed an in silico multiepitope peptide vaccine targeting the OprF and OprE proteins of Pseudomonas aeruginosa, which are conserved across various serogroups and phenotypically stable within biofilms. While the computational predictions regarding immunogenicity, antigenicity, and safety were promising, no experimental validation has been reported to date. Therefore, extensive preclinical and clinical studies are still required to confirm its real-world efficacy (77). Currently, more clinical trials are underway to assess the safety and immunogenicity of peptide vaccine candidates against *P. aeruginosa*. If successful, these vaccines could provide much-needed protection for high-risk individuals, particularly those with underlying medical conditions or undergoing invasive procedures.

### 3.4 Enterococcus faecium

*Vancomycin-resistant Enterococcus faecium* (VRE) has emerged as a significant healthcare-associated pathogen, causing a range of serious infections, including bloodstream infections, surgical site infections, and endocarditis. The increasing prevalence of VRE, coupled with its resistance to multiple antibiotics, has necessitated the development of novel therapeutic strategies (99).

Peptide vaccines provide a new potential strategy for the prevention of VRE infections. These vaccines may target surface proteins, such as Penicillin-Binding Protein 5 (PBP5), which is necessary for the cell wall strength and stability, inducing specific immune responses neutralizing bacteria, and preventing their biofilm formation. Since this protein is an important metabolic target for beta-lactam antibiotic resistance, peptide vaccines against its epitopes can be a preventive measure (100). Additionally, this protein’s multiepitope vaccine with B-cell and T-cell epitopes can elicit both humoral and cellular immune responses, which Dey et al. evaluated in silico, and were promising for experimental testing. Humoral immunity, mediated by antibodies, can neutralize VRE and promote its clearance by phagocytic cells. Cellular immunity, mediated by T-cells, can eliminate infected cells and provide long-lasting protection (79). By enhancing immune system recognition and overcoming immune evasion mechanisms, peptide vaccines have the potential to improve outcomes for patients infected with VRE. Although preclinical and clinical research is needed for a vaccine against this bacterium, they represent a promising strategy to combat this challenging pathogen.

### 3.5 Klebsiella pneumoniae

Carbapenem-resistant Klebsiella pneumoniae (CRKP) has emerged as a major global health threat, causing severe infections such as pneumonia, bloodstream infections, and urinary tract infections (UTIs). The increasing prevalence of CRKP, coupled with its resistance to multiple antibiotics, has led to significant morbidity and mortality (101, 102).

Unfortunately, there is no effective vaccine available against *Klebsiella pneumoniae* (103). Peptide vaccines are considered promising approaches for fighting CRKP infections. Major candidates for peptide-based vaccines against CRKP include outer membrane proteins (OMPs) such as OmpA which are involved in bacterial survival and virulence. In a study conducted by Liao et al., was produced a peptide vaccine to target the OmpA-OmpW-FepA combination protein and then administered that vaccine to mice. Induced by such antigens, the peptide vaccine confers the capabilities of opsonization of bacteria by antibodies; they make them more amenable to clearance by an immune cell and evoke IgG antibodies above any other tested immunoglobulins (81). These peptide vaccines may be used against CRKP, which will improve the outcomes. However, we need more studies to test the safety and efficacy of these peptide vaccines.

### 3.6 Acinetobacter baumannii

*Acinetobacter baumannii* is a gram-negative bacterium with increasing trends toward multidrug resistance, which has constituted a severe health threat worldwide during recent years. This microorganism is thus quasi-invincible due to its ability to form biofilm and its resistance to most known antibiotics, including last-resort antimicrobials including colistin (104).

One of the most interesting strategies to overcome the problems caused by these bacteria is peptide vaccines that important approach is in the targeting of biofilm-related proteins such as Ata, FilF, and Nucab. They are important in bacterial adhesion, biofilm development, and immune evasion (105). Ren et al. developed peptide vaccines targeting these antigens, which inhibit biofilm formation, reduce bacterial colonization, and accelerate immune clearance. In addition, this study developed a peptide vaccine that generated opsonizing antibodies capable of promoting phagocytosis and clearance of developing bacterial infections. This is especially crucial in the setting of chronic infections, in which bacteria can avoid host defenses (86). Peptide vaccines are in progress to combat A. baumannii and improve patient outcomes. They are a potentially powerful weapon against this ornery bug. Many need to be further tested for safety and effectiveness.

## 4 Preclinical and clinical evaluation of peptide vaccines against bacterial infections

Significant progress has been made in the development of peptide vaccines for bacterial infections, and promising results have been obtained from both preclinical and clinical studies.

### 4.1 Animal models

Mice, non-human primates, and other animal models are invaluable for assessing immunogenicity and efficacy of peptide vaccines. Such models enable studies to study the induction of particular immune responses, such as T-cell activation, antibody production and cytokine profiles. For bacterial pathogens, effector-encoded peptide vaccines have shown promise in animal models, including for organisms such as *Staphylococcus aureus*. *S. aureus* peptide vaccines reduce bacterial load and improve survival in animal models of infection (65, 106). While animal models provide valuable insights, it is important to acknowledge their limitations. Differences in immune responses between animals and humans can impact the interpretation of preclinical data. Therefore, studies must carefully consider the selection of appropriate animal models and how to apply findings to human populations (107).

### 4.2 Clinical trials

The transition from preclinical studies to clinical trials is a critical step in the development of peptide vaccines. Phase I clinical trials focus on assessing the safety and tolerability of the vaccine in a small group of healthy volunteers. Phase II trials involve larger groups of participants and aim to determine the optimal dose and evaluate the vaccine’s effectiveness. Phase III trials, the final stage of clinical development, involve large-scale randomized controlled trials to confirm the vaccine’s efficacy and safety in diverse populations. Several peptide-based vaccines targeting bacterial infections are currently undergoing clinical trials, which were mentioned earlier. For example, peptide vaccines for *Pseudomonas aeruginosa* have shown promising safety profiles in Phase I trials. Ongoing Phase II trials are evaluating their immunogenicity and efficacy in larger populations. While these early-phase clinical trials have shown encouraging results, challenges remain in advancing peptide vaccines to Phase III trials. Factors such as peptide stability, immunogenicity, and the development of effective adjuvants and delivery systems are crucial for generalizing preclinical findings into clinical applications (108).

## 5 A Critical appraisal of the safety, stability, and manufacturability of peptide vaccines

### 5.1 Safety considerations

Peptide vaccines have unprecedented safety over traditional vaccines because they are well-defined in their composition. However, the potential risk of autoimmune reactions and off-target effects is a cautious approach. If administered, peptides are highly homologous with host proteins, autoimmunity might develop. The risk of cross-reactivity has been mitigated through careful epitope selection strategies that include targeting sequences with minimal structural similarity to self-antigens. Rigorous preclinical testing — including evaluation of the long-term immune response and assessment of delayed adverse events — is important in these discussions to ensure safety (109). However, advanced bioinformatic tools and structural analyses may also be used to detect peptides with low sequence homology to host proteins. This will lower the possibility of autoimmune reactions (110). However, if not carefully characterized, off-target effects where the immune response targets unrelated strains of bacteria and other commensal microbiota can also pose potential safety issues. Utilizing pathogen-specific peptide sequences and limiting structural similarities with commensal bacteria might mitigate these dangers (111, 112).

### 5.2 Enhanced stability for improved logistics

Peptide vaccines provide significant logistical advantages in stability and storage, particularly in resource-limited environments. Unlike live-attenuated or inactivated vaccines, which often cause stringent cold-chain logistics (113), synthetic peptides exhibit greater stability at room temperature or require only moderate refrigeration. Lyophilization (freeze-drying) further extends shelf life, simplifying distribution and minimizing associated costs. Recent studies demonstrate the viability of specific peptide formulations under diverse storage conditions for extended durations. It’s particularly helpful for deploying them in remote regions (114).

### 5.3 Simplified manufacturing and scalability

Peptide vaccines benefit from streamlined manufacturing processes compared to conventional vaccines. Unlike traditional methods rely on pathogen cultivation, peptide vaccines can be produced through chemical synthesis or recombinant DNA technology. This approach gives greater control over production, which helps achieve high purity and consistency, reduces costs, and speeds up vaccine development. However, scaling up peptide synthesis presents challenges, including potential batch-to-batch variability and high cost influenced by peptide length and complexity. Advancements in automated synthesis techniques and cost-effective production methods are addressing these limitations, which pave the way for wider use and large-scale production of peptide vaccines (108, 115, 116).

## 6 Future directions in peptide vaccine development for bacterial infections

Peptide vaccines are promising tools for the prevention and management of bacterial infections, with ongoing advances in bioinformatics, immunology, and delivery technologies forming the future of their development.

### 6.1 Innovations in peptide design; bioinformatics and artificial intelligence

Due to the development of immunoinformatic tools, the implementation of computational biology has primarily eased the way in the advancement of peptide vaccine development. High-throughput sequencing and structural biology data can be integrated by these technologies to map the immunogenic bacterial epitopes. Using these tools, researchers can predict B-cell and T-cell epitopes, model peptide–MHC and peptide–TCR interactions, and determine binding affinities and epitope stability. Machine learning can help guide this step by finding conserved peptides across multiple strains of bacteria to develop vaccines capable of eliciting long-lived and specific immune responses (117–119).

In recent years, artificial intelligence (AI) has played a transformative role in vaccine design by streamlining several key steps such as antigen selection, epitope prioritization, and adjuvant discovery. Machine learning and deep learning algorithms analyze genomic sequences, protein structures, and immune system interactions to assess immunogenicity and optimize candidate peptides. These AI-driven methods not only reduce the time and cost of vaccine development but also improve precision by integrating emerging technologies like single-cell omics and synthetic biology. Despite challenges such as data heterogeneity and interpretability of models, the incorporation of AI into vaccine research represents a promising approach to accelerate the design of safe and effective peptide vaccines against a broad range of infectious diseases (120).

### 6.2 Personalized peptide vaccines

**Figure 2:**
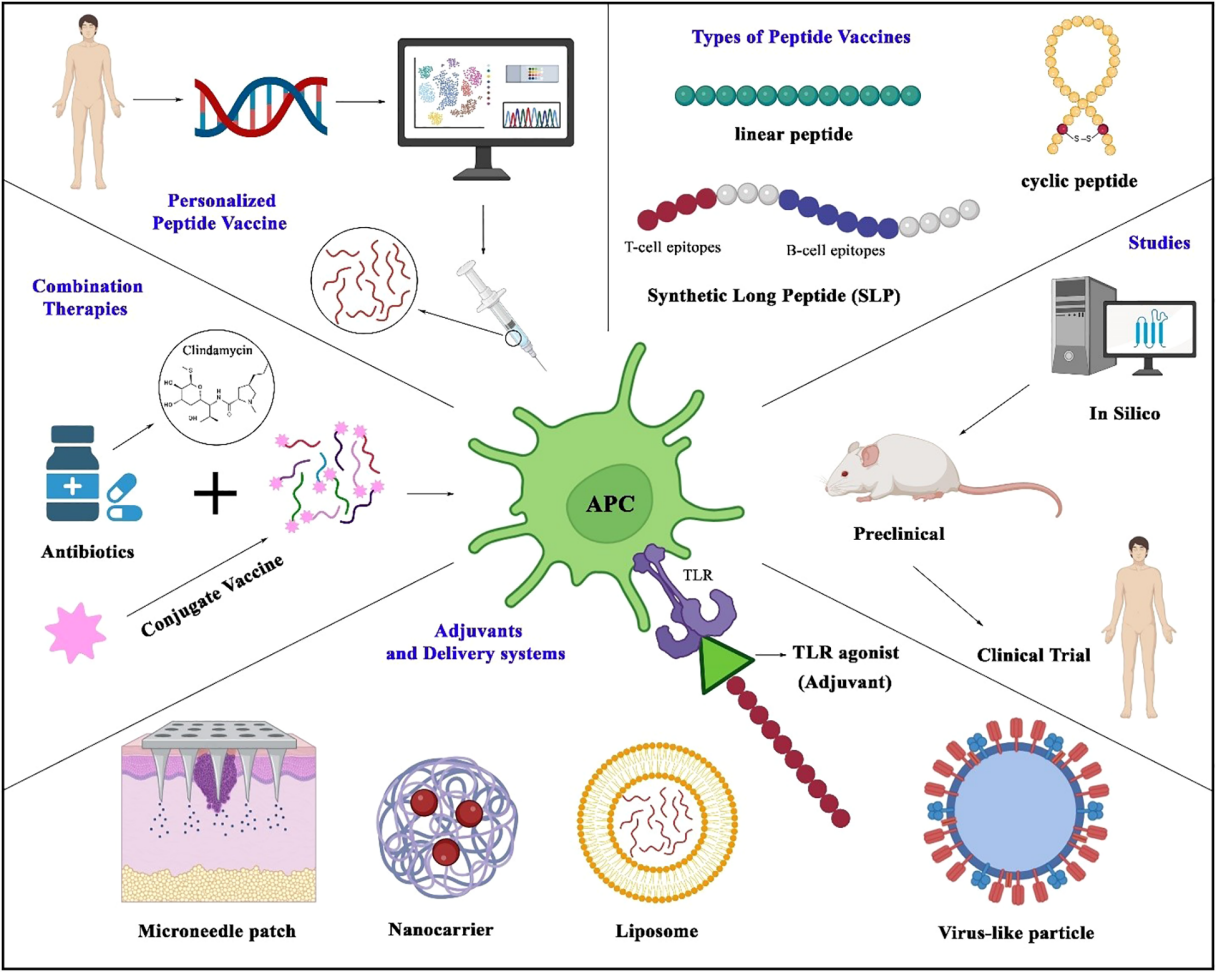
Strategies in peptide vaccine design and application. The figure illustrates key approaches in developing peptide vaccines, centered around antigen-presenting cells that process peptides to activate T-cell immunity. Represented vaccine modalities include linear peptides, cyclic peptides, and synthetic long peptides (SLPs) incorporating both B- and T-cell epitopes. Genomic advances enable personalized vaccines tailored to individual genetic profiles. Delivery platforms (liposomes, nanocarriers, microneedle patches, virus-like particles) and adjuvants (e.g., TLR agonists) enhance immunogenicity. The development pipeline spans in silico epitope prediction, preclinical testing, and clinical trials. Emerging strategies—such as antibiotic-conjugated peptide vaccines—highlight combinatorial approaches to boost efficacy and counter antimicrobial resistance.

The concept of personalized medicine is being applied more often to vaccine development. Differences in immune response among individuals, often due to genetic differences in HLA allele distribution, influence peptide presentation and recognition. Tuning frequencies for specific human genetic profiles can rescue vaccine efficacy and reduce their unwanted effects. This is especially useful for high-risk groups, including healthcare workers and people with immunodeficiency (**Figure 2**) (121).

### 6.3 Next-generation delivery systems

Delivery System of the Next Generation Improving peptide stability and immunogenicity by optimizing vaccine delivery systems is of great significance. Innovative approaches for stable, efficient delivery of peptide vaccines are emerging, such as with virus-like particles (VLPs) and microneedle patches (**Figure 2**). Virus-like particles serve as efficient delivery systems for peptide vaccines by mimicking the structure of viruses while lacking genetic material, allowing safe presentation of antigens to immune cells. VLPs have been modified to present peptide epitopes on their surface to promote antigen uptake and immune activation. This approach has shown promising efficacy against preclinical models and is currently being evaluated for various infectious diseases (10, 122). Microneedle patches penetrate the outer layers of the skin and deliver their payloads—such as peptide antigens—directly to dendritic cells in the dermis in a minimally invasive manner. By targeting these key antigen-presenting cells, microneedles facilitate robust immune activation. Moreover, due to their ease of administration, pain-free application, and potential for self-use, microneedle technologies significantly improve vaccine accessibility and compliance, particularly in low-resource settings (10, 123).

### 6.4 Combination therapies

As shown in **Figure 2**, combining peptide vaccines with antibiotics or other vaccines can enhance their efficacy and expand their activity. For infections resistant to antibiotics, combining peptide vaccines with them can reduce bacterial load and limit the rise of drug-resistant strains (14, 124). Additionally, combining peptide vaccines with other vaccine platforms, such as conjugate vaccines, can provide broader immune coverage against complex pathogens. In this regard, Laura L. Hammitt and colleagues conducted a clinical trial based on a protein-based pneumococcal vaccine (dPly/PhtD) containing pneumolysin toxoid and pneumococcal histidine triple protein D (62).

### 6.5 Harnessing the power of immunomodulation

Combining peptide vaccines with immunomodulators, such as checkpoint inhibitors, can amplify immune responses. Checkpoint inhibitors can enhance T-cell responses, particularly beneficial for chronic bacterial infections that require a strong cell-mediated immune response. Such combination therapies may allow for lower antibiotic doses, which could reduce side effects and the risk of antibiotic resistance (125, 126).

## 7 Conclusion

Peptide vaccines offer a promising approach to combat bacterial infections. By targeting specific antigens, these vaccines can induce targeted immune responses, enhancing the body’s ability to recognize and eliminate bacterial pathogens. Key advantages include rapid production, cost-effectiveness, and suitability for immunocompromised individuals. However, challenges such as improving immunogenicity, stability, and delivery methods remain. Ongoing research is focused on optimizing peptide design, developing advanced delivery systems, and exploring combination therapies to enhance vaccine efficacy. With continued advancements, peptide vaccines have the potential to become a crucial tool in the struggle against antibiotic resistance and emerging infectious diseases.
